# Unravelling Twin Births in German Holstein Cows: Phenotypic Associations, Genetic Analysis and Potential Underlying Genes and Hormones

**DOI:** 10.1111/jbg.70012

**Published:** 2025-08-21

**Authors:** Laura Hüneke, Hatem Alkhoder, Dierck Segelke, Georg Thaller, Christin Schmidtmann

**Affiliations:** ^1^ IT Solutions for Animal Production (vit) Verden Germany; ^2^ Institute of Animal Breeding and Husbandry Christian‐Albrechts‐University Kiel Kiel Germany

## Abstract

Twin births in dairy cattle are rare but present significant challenges for animal welfare, as both the health of the cow and the calves are affected. This causes economic losses, which prompts breeders to select against twin births and identify associated risk factors. This study examines the phenotypic relationship between milk yield, fertility traits and twin births in German Holstein cattle using a large, population‐wide dataset. GEBV correlations for twin births, milk production and fertility traits were estimated. Genome‐wide association studies (GWAS) were conducted for calving numbers 1–3 in order to explore the genetic background in more detail. The twin birth rate showed a strong phenotypic association with milk production and a moderate phenotypic association with the timing of successful insemination. However, GEBV correlations were low: 0.04 with milk yield and −0.10 to 0.01 with fertility traits. GWAS revealed two potential candidate genes on BTA11: *LHCGR* and *FSHR*, which encode receptors for LH and FSH, two hormones crucial to estrus. In contrast to the first calving, significantly associated regions on BTA5 and BTA25 were found in calving numbers 2 and 3. This study demonstrates the interaction between genotype and environment, concluding that a genetic predisposition for twin births, in combination with a favourable endocrine state (environment), increases the likelihood of twin births.

## 1 Introduction

Cattle are a monotocous species, meaning that usually one calf is born per birth. However, multiple newborns per birth are possible, but come along with increased birth difficulties and health issues for both cows and the calves (Vinet et al. 2012). The average twinning rate in cattle varies among populations and breeds, with beef cattle mostly having lower twin birth rates compared to dairy cattle (Rutledge 1975; Fricke 2001). In Holstein cattle, twin birth rates are estimated to be 1.9%–4.2% (Ghavi Hossein‐Zadeh et al. 2009; Cabrera and Fricke 2021; Kirkpatrick and Berry 2025), but some studies also report herds having up to 10% twin births (Kinsel et al. 1998). A phenomenon that all studies report is that twin birth rate depends on the parity. The lowest twinning rates are observed in the first parity cows, whereas the twinning rate in higher parities is often more than doubled (Johansson et al. 1974; Beerepoot et al. 1992; Widmer et al. 2021). A key distinction between the first and subsequent parities is that cows in higher parities are lactating at the time of insemination. This physiological state appears to influence the likelihood of multiple ovulations, which account for 95% of all twin births (Silva del Río et al. 2006). In contrast, only 5% of all twin births are monozygotic, resulting from spontaneous embryonic cleavage.

Several studies report that a higher milk production is associated with an increased likelihood of double ovulation and thus twinning rate (Nielen et al. 1989; Kinsel et al. 1998; Fricke and Wiltbank 1999). The underlying mechanism involves a complex interplay of multiple hormones that regulate the development of a dominant follicle. Among these, the concentration of progesterone, a steroid hormone, affects the incidence of double ovulations (Martins et al. 2018; Carvalho et al. 2019). In high‐yielding cows, increased metabolic rates and enhanced blood flow have been shown to significantly affect steroid metabolism in the liver, where circulating blood is efficiently cleared of steroids during hepatic circulation (Parr et al. 1993; Wiltbank et al. 2000). Even though this relationship explains the observed phenotypic correlation between milk production and twinning rate, genetic correlations are low (Ron et al. 1990; McGovern et al. 2021; Hüneke et al. 2025). Deeper analysis of the genetic architecture of the trait twin birth revealed two major possible candidate genes influencing the trait: *LHCGR* and *FSHR*, both lying on chromosome 11 (Widmer et al. 2021; Kirkpatrick and Berry 2025). These genes are encoding receptors for the luteinizing hormone/choriogonadotropin (LH/CG) and the follicle stimulating hormone (FSH; Widmer et al. 2021; Kirkpatrick and Berry 2025). However, genetic correlations between twin births and fertility traits are low (McGovern et al. 2021; Hüneke et al. 2025).

The aim of this study was to examine the relationship between milk production, fertility traits, and twin births in German Holstein cattle in greater detail. To highlight the differences between first parity and later parities, as well as between non‐lactating and lactating cows, twin births were analysed across different parities. This investigation combined phenotypic analyses with genetic correlations and genome‐wide association studies (GWAS) approaches.

## 2 Material and Methods

### 2.1 Data Preparation

Data records of cows born between 2000 and 2023 were available from the routine genetic evaluation of German Holstein, including calving records (date of the calving, singleton/twin), insemination data (date of successful insemination, AI bull, previous inseminations) and milk yield (305d yield). General plausibility checks and preparation were made. Only records from Holstein cows were used. If the sire or dam was unknown, data was excluded. Calvings from dams with an age at first calving below 19 months as well as calvings from embryo transfer were excluded. Finally, one record per calving event was kept with the information if the dam had a singleton or multiple calves at this calving event. This trait was binary coded (twin birth yes/no, 0/1) and triplets were counted as twin births due to their low prevalence (< 0.005%).

### 2.2 Phenotypic Analyses

The phenotypic relationship between milk yield (305 days yield) and twin birth rate was analysed for calving years from 2015 to 2023. Since the mean milk yield per lactation differs, only the milk yield in first lactation was considered, along with the twin birth rate of the subsequent calving (2nd calving). Cows were grouped based on their 305 days milk yield into eight groups from < 6000 to > 12,000 kg. For each group, the mean twin birth rate of the 2nd calving was calculated. In total, 3,518,349 cows were analysed, and each group contained between 58,906 (> 12,000 kg) and 825,173 (**≥** 8000 kg) cows. The number of animals for each group is given in Table S1.

The days in milk (DIM) when the successful insemination (leading to the next calving) occurred was calculated as the number of days since the start of the lactation (calving) until the day of the successful insemination. Consequently, only calvings 2, 3 and 4 were analysed. Since the mean DIM for successful inseminations showed no significant variation over the past 20 years, the entire dataset from 2000 onward was used. DIM values were considered valid if they ranged between 10 and 305 days. Cows were grouped based on their DIM at the time of successful insemination, using 21‐day intervals to reflect the typical length of the estrous cycle. The peak ovulation day (e.g., Day 21, 42, 63) was positioned at the midpoint of each interval. The DIM‐groups contained between 5463 (DIM 10‐31, calving number 4) and 1,425,874 (DIM 73‐93, calving number 2) cows. The number of animals for each group is given in Table S2. The mean twin birth rate was calculated for each group.

### 2.3 GEBV Correlations

Genomic estimated breeding values (GEBVs) for twinning rate were calculated using a single‐step SNPBLUP model as described by Hüneke et al. (2025). Twinning was modelled as two genetically correlated but distinct traits: (1) twin births in first parity, and (2) twin births in later parities. This separation was justified by the observed genetic correlation of 0.88 between first and later parities, whereas the correlation between the higher parities themselves was considerably higher (0.99) (Hüneke et al. 2025). A higher GEBV indicated a greater genetic disposition for twin births. GEBV correlations between the twinning rate traits and other breeding goal traits were calculated for 327,478 genotyped cows born in 2022 and 2023. The selection of two birth years prohibits bias through genetic trends. Correlations were calculated for the most important traits (milk, longevity, health, calving traits) and all fertility traits that are estimated during the German routine breeding value estimation (vit 2024).

### 2.4 Genome‐Wide Association Studies

Genotypes (45,613 SNP markers) and pedigree data of German Holstein cows born between 2018 and 2024 were obtained from the routine genetic evaluation in April 2024 (vit 2024). The SNP positions and alleles were mapped to the bovine reference genome ARS‐UCD1.2. To account for potential parity‐specific genetic effects, separate GWAS were conducted for calving numbers 1–3. Due to computation capacity reasons, a random sample of 100,000 animals for the 1st calving number was chosen. For the 2nd and 3rd calving numbers, 141,523 and 67,518 animals were included in the analysis, respectively. The sex chromosomes were not considered in the analysis. Quality control of data was carried out, and SNPs with a minor allele frequency < 1% were removed, so that 42,587 SNPs remained.

Single trait GWAS were performed for twin births in calving numbers 1–3 using GCTA (Yang et al. 2011) and the following single SNP regression mixed linear model:

y=μ+Xb+Zg+Wv+e

where y was the vector of the phenotypes (twin birth (0/1)); μ was the overall mean; b was the vector of the fixed effects sexed semen, herd, and year‐season with the respective incidence matrix X; g was the vector of polygenic effects with $g\sim N\left(0,G\sigma_{g}^{2}\right)$, where G represented the genomic relationship matrix and $\sigma_{g}^{2}$ was the polygenic additive variance; Z was the respective incidence matrix of g; v was the vector of the additive effect of the tested SNP (coded as 0, 1, 2) with its incidence matrix W; and e was the vector of random residuals. Genetic variants were considered to be significantly associated with the trait twin births at a Bonferroni corrected threshold of *p* < 1.17 × 10–6 [(0.05/42,587), $-\log_{10}\left(p\right)\approx 5.93$]. In addition, a less conservative threshold for suggestive associations was set to *p* < 2.35 × 10–6 [(1/42,587), $-\log_{10}\left(p\right)\approx 4.63$].

## 3 Results

### 3.1 Phenotypic Analysis

**FIGURE 1:**
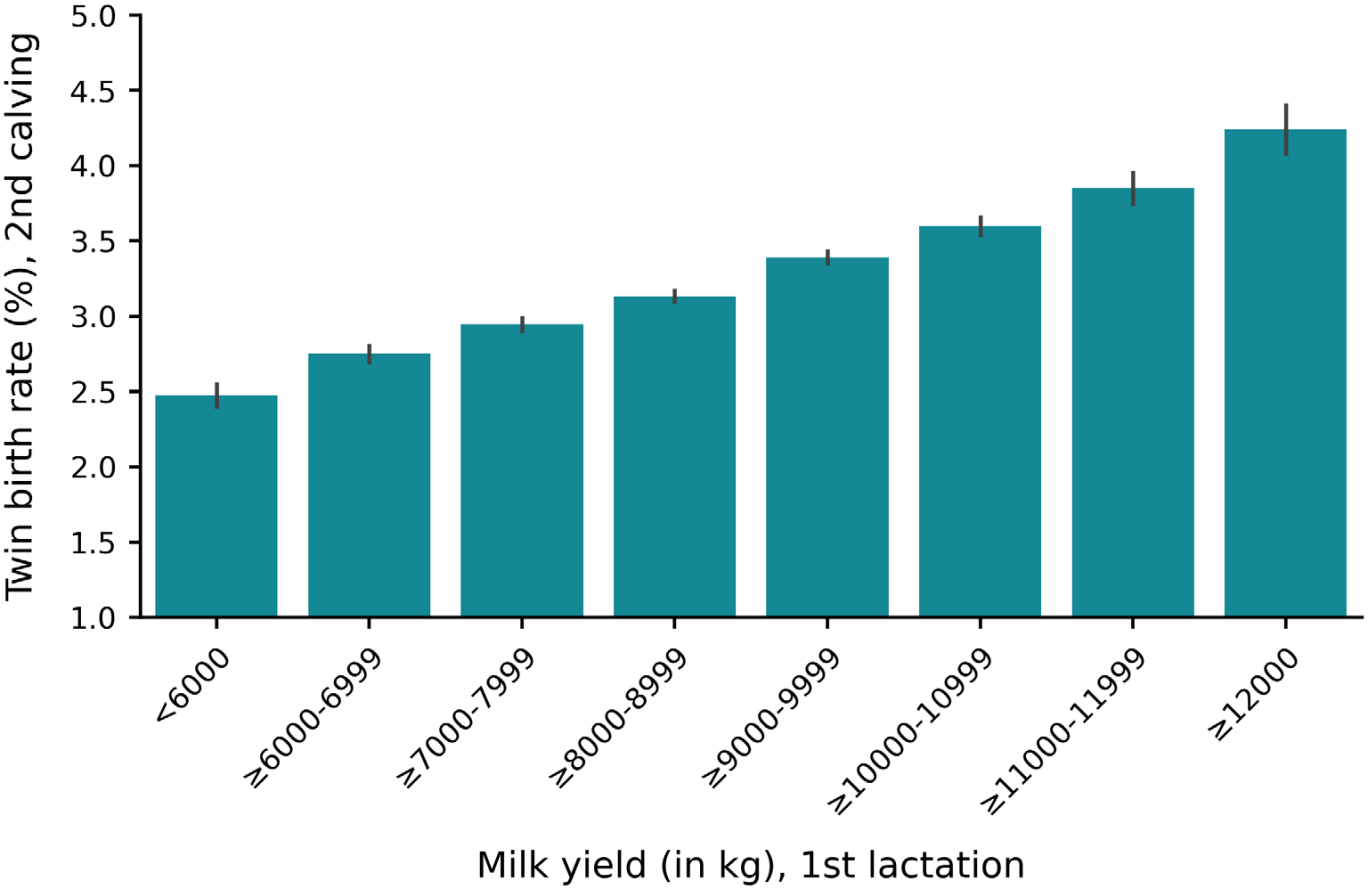
Milk yield (in kg) in the 1st lactation and mean twin birth rate (%) of the 2nd calving. The black lines represent the 95% confidence interval. [Colour figure can be viewed at wileyonlinelibrary.com]

Figure 1 shows the phenotypic relationship between the milk yield in the previous lactation and the average twin birth rate of the subsequent calving within the defined cow groups. The frequency of twin births tends to be higher when milk yield in the previous lactation is increasing. The difference in the average twin birth rate between low and high yielding cows is more than 1.5%.

**FIGURE 2:**
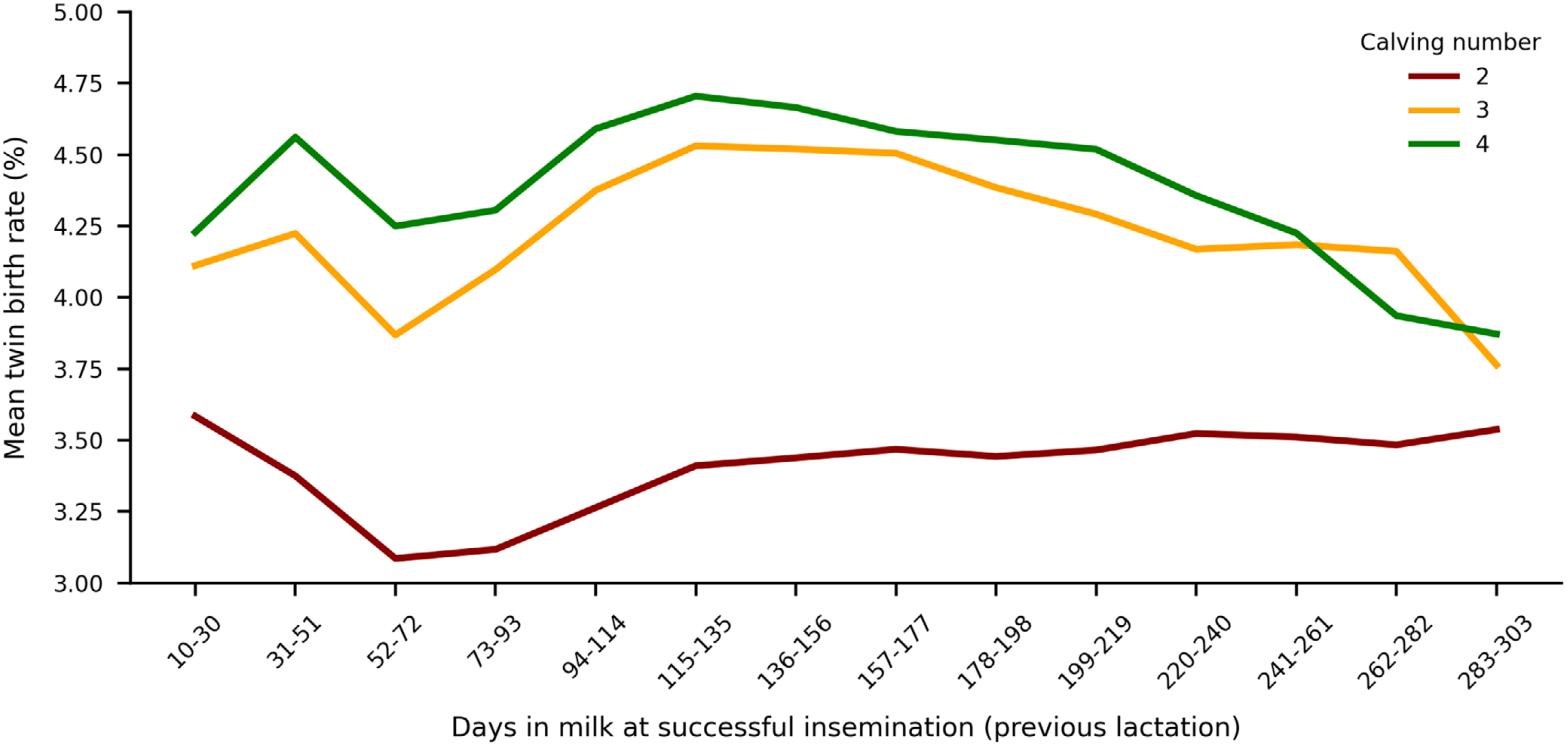
Mean twin birth rate (%) of calving number 2, 3 and 4 by days in milk (DIM) at successful insemination in the previous lactation. [Colour figure can be viewed at wileyonlinelibrary.com]

The relationship between the DIM on which the successful insemination took place, and the average twin birth rate for parity 2, 3 and 4 is illustrated in Figure 2. The data reveal distinct patterns depending on parity number. The twin birth rate is notably lower in 2nd parity and relatively similar in parity 3 and 4, but usually 0.25% higher in parity 4. The highest twin birth rate in parity 2 occurs during the early lactation phase (10–30 DIM). A sharp decline is observed, reaching the lowest rate at 52–72 DIM, after which the rate stabilises at a level of ≈3.5%. Parities 3 and 4 also show a decline of mean twin birth rate at DIM 52‐72. In contrast to parity 2, the rates in both groups peak during 115–156 DIM and then gradually decline towards the later stages of lactation.

### 3.2 GEBV Correlations

**FIGURE 3:**
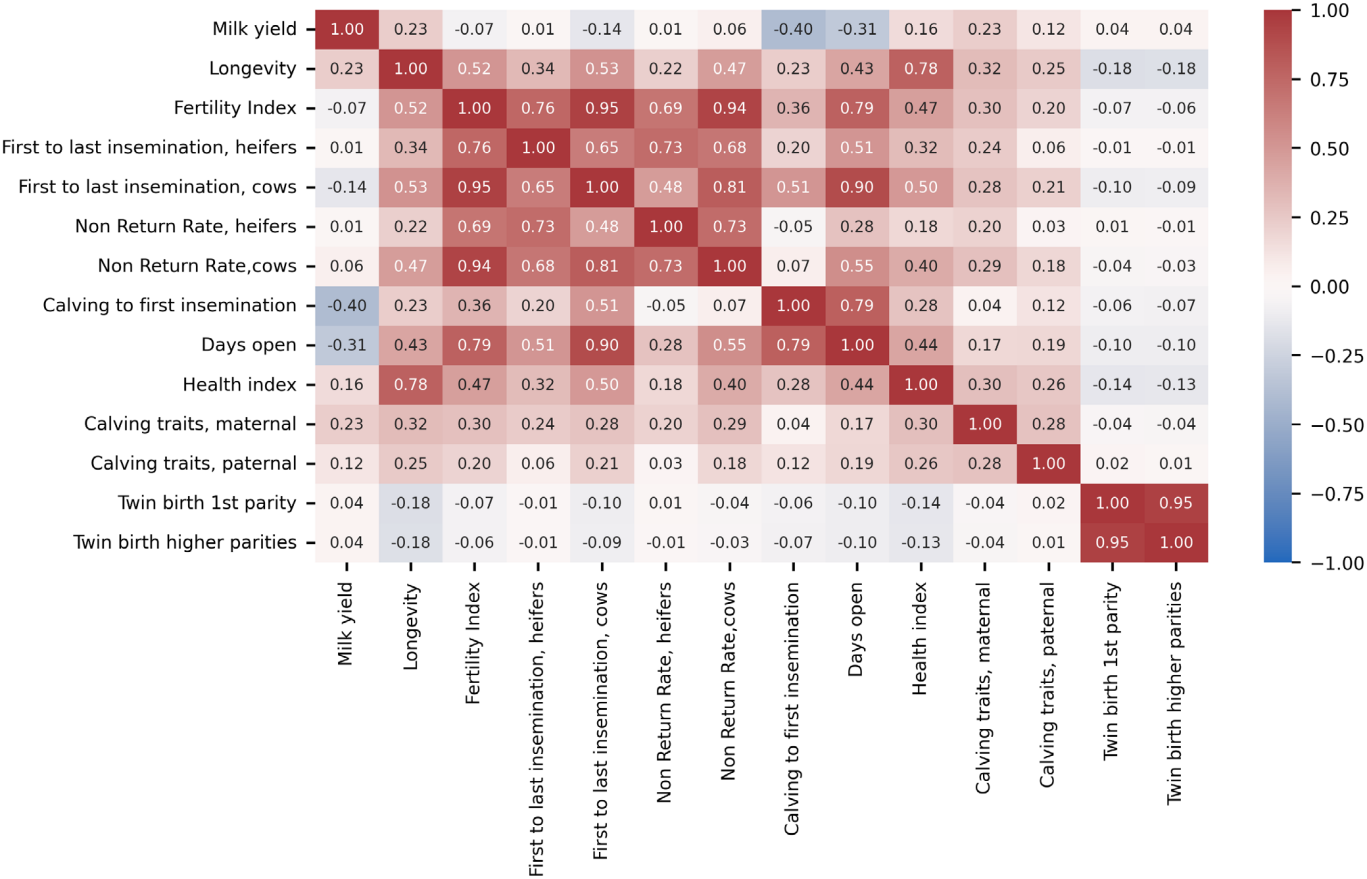
Correlation heatmap of genomic estimated breeding values (GEBVs). [Colour figure can be viewed at wileyonlinelibrary.com]

The heatmap in Figure 3 displays correlations among GEBVs for various traits. Of particular interest are the correlations involving the GEBVs for twin birth rate in the 1st parity (‘Twin birth 1st parity’) and in higher parities (‘Twin birth higher parities’). These GEBVs are highly correlated (*r* = 0.95). The correlations between twin birth GEBVs and all other traits are very weak and mostly close to zero. However, the strongest correlation to twin births was found for longevity, showing a negative correlation of −0.19 to twin births in 1st parity and −0.18 to twin births in higher parities. Correlations with the health index (including direct health traits like mastitis, digital dermatitis and retained placenta) are somewhat lower (*r* = −0.14 and −0.13). Among the fertility traits the strongest correlation of −0.09 was observed for days open and first to last insemination of cows. For the traits first to last insemination and Non‐Return Rate GEBVs are calculated for heifers and for cows. For both traits the correlation with the respective trait for cows is slightly higher.

### 3.3 Genome‐Wide Association Studies

**FIGURE 4:**
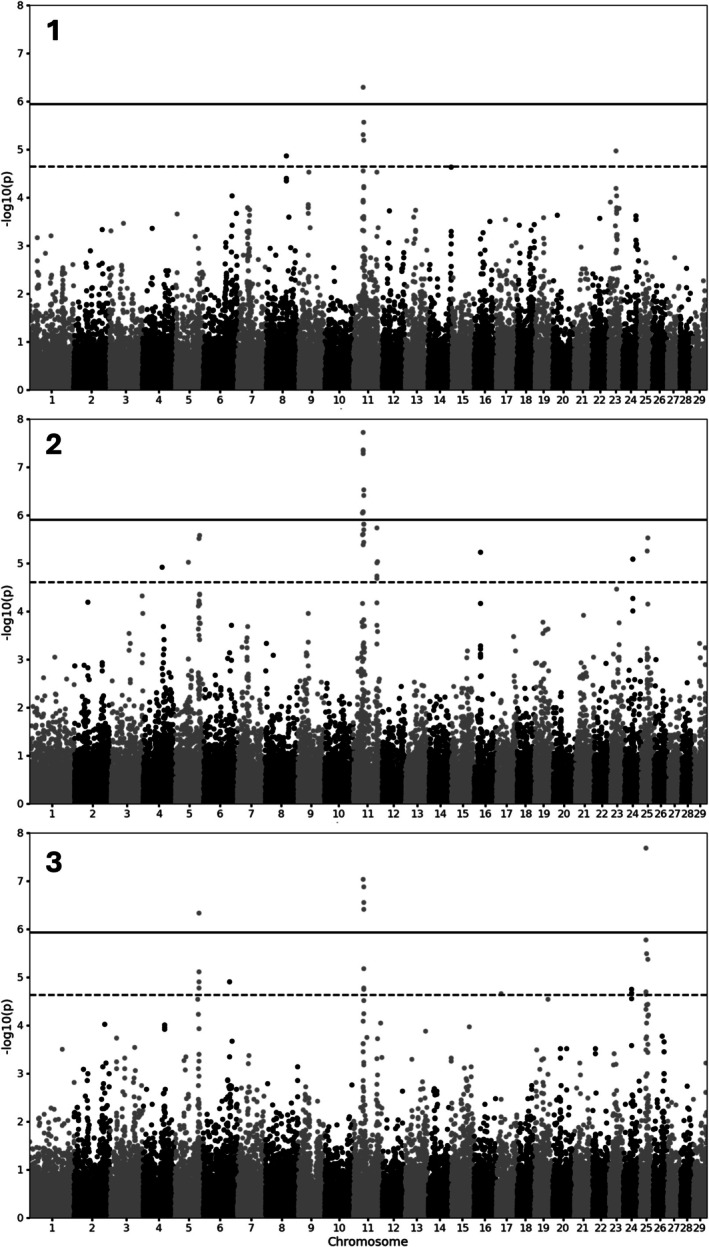
Manhattan plots showing −log_10_(*p*) of GWAS for twin births in parity 1, 2 and 3. The dashed line indicates the suggestive threshold at −log_10_(*p*) = 4.63, whereas the solid line represents the genome‐wide threshold at −log_10_(*p*) = 5.93.

The GWAS for twin births for calving number 1, 2 and 3 reveals multiple significant SNPs (Figure 4). Although for calving number 1 only two SNPs pass the genome‐wide threshold, calving numbers 2 and 3 show several SNPs above this threshold. For each calving number, a peak at BTA11 is visible. Calving numbers 2 and 3 also display peaks at BTA5 and BTA25. In the GWAS for calving number 3, SNPs within these peaks surpass the genome‐wide threshold. All SNPs significantly associated with twin births are listed in Table 1. Additionally, SNPs exceeding the suggestive threshold are provided in Table S3.

**TABLE 1:** Significantly associated SNPs with twin births.

Chromosome	Position (bp)	SNP	Effect	*p*	Calving number
5	106,895,411	ARS‐BFGL‐NGS‐118796	0.01400	4.60e‐07	3
11	28,689,341	ARS‐BFGL‐NGS‐22001	−0.00632	8.99e‐07	2
11	29,060,501	ARS‐BFGL‐NGS‐55891	−0.00677	9.06e‐07	2
11	29,237,085	ARS‐BFGL‐NGS‐14086	−0.00942	3.00e‐11	2
11	29,333,199	ARS‐BFGL‐NGS‐103581	−0.00982	2.29e‐12	2
11	29,574,227	ARS‐BFGL‐NGS‐62651	−0.00896	4.35e‐08	2
11	29,601,504	BTB‐01522198	−0.00895	4.59e‐08	2
11	29,624,765	ARS‐BFGL‐NGS‐5463	−0.00891	5.24e‐08	2
11	29,904,900	ARS‐BFGL‐NGS‐118268	−0.00250	4.97e‐04	1
11	29,977,957	ARS‐BFGL‐NGS‐86581	0.00632	1.90e‐08	2
11	29,977,957	ARS‐BFGL‐NGS‐86581	0.00910	9.34e‐08	3
11	30,282,753	ARS‐BFGL‐NGS‐35518	0.00600	8.46e‐07	2
11	30,804,812	BTB‐00489432	−0.00558	8.57e‐07	2
11	30,916,713	ARS‐BFGL‐NGS‐82216	−0.00588	2.98e‐07	2
11	30,942,534	BTB‐01872902	−0.00585	3.92e‐07	2
11	31,004,983	ARS‐BFGL‐NGS‐83866	−0.00871	4.22e‐15	2
11	31,004,983	ARS‐BFGL‐NGS‐83866	−0.00868	2.76e‐07	3
11	31,096,059	BTA‐99659‐no‐rs	−0.00809	1.78e‐13	2
11	31,096,059	BTA‐99659‐no‐rs	−0.00887	1.33e‐07	3
11	31,274,292	Hapmap45323‐BTA‐90907	0.00298	5.04e‐04	1
11	31,369,325	UA‐IFASA‐9493	−0.01060	1.30e‐18	2
11	31,369,325	UA‐IFASA‐9493	−0.00925	3.87e‐07	3
11	31,441,793	ARS‐BFGL‐NGS‐111198	−0.00747	7.28e‐09	2
25	20,683,225	ARS‐BFGL‐NGS‐42285	−0.00849	2.09e‐08	3

*Note:* Positions are based on the ARS‐UCD1.2/bosTau9 assembly.

The region on BTA11, where the most significantly associated SNPs are located, contains two genes, which are possible candidate genes for twin births: *LHCGR*, encoding the luteinizing hormone/choriogonadotropin receptor (chr11:30,977,805–31,040,344) and *FSHR*, encoding the follicle stimulating hormone receptor (chr11:31,255,649–32,450,537).

## 4 Discussion

### 4.1 Milk Production Increases Twinning Rate

Our results showed that the milk production during the lactation in which the insemination occurred is associated with an increased twinning rate. The difference in twinning rate between high and low yielding cows is 1.5%, which is remarkable for a trait with a low prevalence. High milk production as a risk factor for increased twinning rate has already been reported in several studies (Kinsel et al. 1998; Fricke and Wiltbank 1999; Lopez, Caraviello, et al. 2005). In a study conducted on a large dataset in the US, the odds ratio for twin births when comparing Holstein cows producing 8000 and 13,000 kg was 1.23 (Schambow et al. 2021). Even though a strong phenotypic relationship exists, the genetic correlation between milk yield and twinning rate is very low, with reported estimates ranging from 0.011 to 0.13 (Ron et al. 1990; McGovern et al. 2021; Kirkpatrick and Berry 2025). In the population analysed in this study, the genetic correlation between milk yield and twinning rate was estimated to be 0.048 for the first parity and 0.062 for the higher parities (Hüneke et al. 2025), confirming the weak genetic link between the two traits. In terms of breeding, the low correlation between the two traits is favourable because milk production is a main breeding goal in Holstein cattle. A strong positive genetic correlation, as one might expect based on the phenotypic association, would have meant that twinning rates would have increased indirectly through selection on high yielding cows. However, the trend in twin births has not increased over the last 20 years, whereas milk production has increased significantly (Widmer et al. 2021; Guinan et al. 2023; Hüneke et al. 2025). Consequently, milk traits and twinning rate do not share a common genetic foundation, suggesting that the observed phenotypic association is most likely influenced by environmental factors, such as the hormonal status of the cow. High producing cows have a lower level of circulating progesterone, affecting follicle development and thus the incidence of multiple ovulations (Lopez, Caraviello, et al. 2005; Martins et al. 2018). Progesterone plays a crucial role on the hypothalamic–pituitary–gonadal axis by exerting negative feedback through the suppression of gonadotropin‐releasing hormone (GnRH) neurons in the hypothalamus. The subsequent reduction in GnRH concentration inhibits the release of LH and FSH from the pituitary gland (Plant 2015). Thus, when progesterone levels are low, as observed in high producing cows, the negative feedback is reduced, allowing LH and FSH concentrations to be less inhibited, which could promote the development of multiple dominant follicles (Lopez, Caraviello, et al. 2005; Lopez, Sartori, et al. 2005; Martins et al. 2018). Consequently, it is not the genetic capacity for milk production itself but rather the resulting hormonal dynamics that impacts the occurrence of twin births.

### 4.2 Hormonal Dependency

The impact of the endocrine state of a cow on the likelihood for multiple ovulations and thus twinning rate is high, as already described above. In addition, the timing of the successful insemination during the lactation has an influence on the likelihood of twin births, as shown in Figure 2. Although twin births generally become more frequent with later insemination, this pattern varies by lactation: the increase is continuous in the second parity, but in the third and fourth parity, the frequency rises only up to around 150 DIM and then declines. The negative energy balance at the beginning of the lactation might have an influence on the twin birth rate, as it has several effects on fertility and the ovary (Wathes et al. 2007). The energy deficit influences liver function and decreases the expression of several hormones, such as insulin‐like growth factor 1 (IGF‐1), which results in slowed follicular growth (Lucy et al. 2001; Wathes et al. 2007). Studies show that energy levels are linked to ovulation incidence, suggesting that as the cow moves further from calving and exits negative energy balance, more energy is allocated towards fertility, increasing the likelihood of multiple ovulations (Kinsel et al. 1998; Echternkamp, Cushman, et al. 2007). However, the effect of the date of the successful insemination (DIM) is relatively small, as the variations in the phenotypic twin birth rate are < 1%.

The GEBV correlations between fertility traits and twin births are slightly negative and close to zero. The strongest correlations were found between first to last insemination (cows) and days open. The German fertility GEBVs are defined in such a way that high GEBVs indicate a desirable breeding trait. This means that a high GEBV for the first to last insemination corresponds to a shorter time span between the first and last insemination. In contrast, high breeding values for twin births indicate a higher likelihood of twin births. The negative correlation thus suggests that as the probability of twin births increases, both the time span between the first and last insemination and the number of days open tend to increase as well. From a breeding perspective, this correlation is viewed positively, as breeding aims to reduce the frequency of twin births. Additionally, these results are consistent with the phenotypic associations shown in Figure 2, which indicate that the twin birth rate increases with increasing DIM. However, it is important to note that the correlations are very low, emphasising the complexity of both traits.

### 4.3 Genetic Influence

GWAS showed significant associations with SNPs on BTA11 in all three calving numbers. Potential candidate genes located in this region are *LHCGR* and *FSHR*, which encode the receptors for essential hormones for the estrus. These two genes were already found in recent studies in Swiss, US and Irish populations (Widmer et al. 2021; Lett and Kirkpatrick 2022; Kirkpatrick and Berry 2025). In addition, also in humans, the homologous region of the two candidate genes *LHCGR* and *FSHR*, located on chromosome 2, was reported to have an effect on twin birth rate (Derom et al. 2006). Interestingly, the pattern of significant SNPs differs between the calving numbers. Although the peak on BTA11 remained constant, two peaks on BTA5 and BTA25 occur in calving numbers 2 and 3. BTA5 was already focused on in the twinner population of the US Meat Animal Research Center (USMARC; Clay Center, NE), a population in the US, which was selected for high twinning rates. Significant QTLs with an influence on ovulation rate were identified by Kappes et al. (2000) and Kirkpatrick et al. (2000). In addition, Allan et al. (2009) identified BTA5 as containing several strong candidate genes involved in follicular and embryonic development, as well as the increase in ovulation rate. Another possible candidate gene on BTA5 is the *IGF1* (insulin‐like growth factor 1), as studies showed significant influence of IGF‐1 on twinning rate (Echternkamp et al. 1990) and even detected a suggestive association with an *IGF1* gene polymorphism (Kim et al. 2009). Surprisingly, this potential candidate gene is not located in the region that was found in this study. The same observation was also made by Lett and Kirkpatrick (2022), who identified significant SNPs at a suggestive level, but not in the region of *IGF1*. No previous reports of significant QTLs and potential candidate genes on BTA25 are available; however, Lett and Kirkpatrick (2022) also found significant associations in this region.

The high level of noise observed in the GWAS might be attributed to environmental influences not accounted for in the analysis and the polygenic nature of the trait, with small effect sizes distributed across many loci, likely exacerbating the challenge of identifying robust associations. The number of significant SNPs, including those surpassing the suggestive threshold, supports the conclusion that twinning is a quantitative trait influenced by multiple genetic regions. Additionally, the variation in associated regions between animals in different parities may indicate that distinct genes are involved in each parity. The GWAS results for the first calving number differ from those of the subsequent calving numbers, whereas the 2nd and 3rd calving numbers show similar patterns. Previous findings showed that the genetic correlation between the first and later parities is lower compared to the genetic correlations observed among higher parities (Weller et al. 2008; Hüneke et al. 2025). In the study by Hüneke et al. (2025), which analysed the same population as in this study, the genetic correlation between the first and later parities was 0.88, whereas correlations among higher parities, such as between the second and third parity, were as high as 0.99. However, the potential candidate genes on BTA11 seem to have the highest influence on twinning, as the associated SNPs are significant in all the analysed calving numbers. This finding aligns with the ‘omnigenic model of complex traits’ proposed by Boyle et al. (2017). According to this model, a few ‘core genes’ play a direct and biologically interpretable role in the trait, as is the case for *LHCGR* and *FSHR*, which are likely to have the strongest effects. However, these core genes explain only a small fraction of the total genetic variance (Manolio et al. 2009). Beyond the core genes, numerous causal loci with small effect sizes contribute to the expression of the trait (Goldstein 2009). These variants may be distributed throughout the genome, often distant from genes with specific functions related to the trait. Boyle et al. (2017) attribute this phenomenon to cell regulatory networks, where any expressed gene could influence the regulation or function of core genes. For complex traits characterised by multiple variants with small effect sizes, sample size significantly impacts the ability to detect rare variants (Visscher et al. 2017). Although the sample size was large compared to other GWAS studies in livestock, analysing the trait as a repeated measure with whole population data could improve the robustness of the results.

### 4.4 Dynamics of Genetics and Hormonal State

Twinning is a quantitative trait influenced by many environmental factors. The wide range of risk factors for twin births reported in the literature, such as parity, breed, season, milk production, drug therapy (Kinsel et al. 1998; Fricke 2001), suggests that the contribution of genetics may be relatively limited compared to environmental influences. Nevertheless, heritability estimates of the twinning rate range between 0.008 and 0.031 (Johansson et al. 1974; Weller et al. 2008; Lett and Kirkpatrick 2018; Hüneke et al. 2025), showing that the trait is heritable. Selection experiments aimed at increasing the twin birth rate, such as those conducted in the USMARC twinner population, demonstrated a rapid rise in twinning rates up to 28.5% (Van Vleck and Gregory 1996; Echternkamp, Thallman, et al. 2007), thereby highlighting the role of the genomic component. Although the phenotype is determined by genotype and environment, the interaction between the two appears particularly relevant for twinning. Our findings showed a strong phenotypic association between milk production and twinning, likely driven by the endocrine status of high‐yielding cows. In parallel, GWAS identified significant associations with two genes involved in female reproduction, suggesting a genotype‐environment interaction. One possible explanation is that cows with a high genetic predisposition for twin births may express higher levels of *LHCGR* and *FSHR* genes, resulting in more receptors that enhance the binding capacity of LH and FSH. When combined with a hormonal state that promotes twinning, characterised by low progesterone and high LH and FSH concentrations, these cows might be even more susceptible to multiple ovulations due to their strong genetic predisposition. Although these genes were also significant in first‐parity cows, twinning rates are much lower at first calving (Beerepoot et al. 1992; Johanson et al. 2001; Hüneke et al. 2025), likely due to the hormonal environment in heifers, which are not lactating at the point of insemination.

Further studies using large‐scale genomic data, including whole‐genome sequencing and transcriptomic analyses, are promising to identify additional key genes and mechanisms. Exploring gene–environment interactions and epigenetic factors may also enhance understanding of the complex genetic architecture underlying twinning in cattle.

## 5 Conclusion

This study highlights the complex genetic and environmental interactions influencing the twin birth rate in dairy cattle. Our results demonstrate a strong phenotypic relationship between milk production and twin birth rate, driven primarily by the endocrine status of high‐yielding cows. The GWAS revealed significant associations with genes critical for female reproduction, suggesting a genetic basis for twinning. However, the interaction between genotype and environment, particularly hormonal factors such as progesterone, LH and FSH levels, appears to play a crucial role in determining the likelihood of multiple ovulations.

## Ethics Statement

The authors have nothing to report.

## Conflicts of Interest

The authors declare no conflicts of interest.

## Supporting information


**Data S1:** jbg70012‐sup‐0001‐Tables.docx.

## Data Availability

Restrictions apply to the availability of the data used in this study. Vit does not own the data but is allowed to work with it under a licence of a material transfer agreement for the current study. Thus, data are not publicly available.
