# Supplementary material for: Unravelling Twin Births in German Holstein Cows: Phenotypic Associations, Genetic Analysis and Potential Underlying Genes and Hormones
Source: J Anim Breed Genet. 2025 Aug 21;143(2):213–22. doi: 10.1111/jbg.70012 (PMC12887153; doi:10.1111/jbg.70012)
Supplement: Supplementary file 1 — Data S1: jbg70012‐sup‐0001‐Tables.docx. [file JBG-143-213-s001.docx]

# Supplementary

Supplemental Table 1: Number of cows per milk yield category, based on analysis shown in Figure 1

| **Milk yield category** | **Number of cows** |
| --- | --- |
| < 6000 | 154,323 |
| ≥ 6000 – 6999 | 364,403 |
| ≥ 7000 – 7999 | 682,868 |
| ≥ 8000 – 8999 | 825,173 |
| ≥ 9000 – 9999 | 663,991 |
| ≥ 10,000 – 10,999 | 369,080 |
| ≥11,000 – 11,999 | 149,462 |
| ≥ 12,000 | 58,906 |

Supplemental Table 2: Number of cows per DIM category and calving number, based on analysis shown in Figure 2

| **DIM category** | **Calving number 2** | **Calving number 3** | **Calving number 4** |
| --- | --- | --- | --- |
| 10 – 30 | 18,665 | 13,328 | 5,463 |
| 31 - 51 | 500,367 | 320,402 | 162,674 |
| 52 – 72 | 1,404,122 | 946,747 | 536,177 |
| 73 – 93 | 1,425,874 | 1,019,297 | 629,015 |
| 94 – 114 | 1,117,888 | 833,602 | 538,536 |
| 115 – 135 | 844,987 | 648,637 | 426,593 |
| 136 – 156 | 638,027 | 496,016 | 330,400 |
| 157 - 177 | 480,251 | 376,833 | 253,163 |
| 178 – 198 | 359,687 | 280,897 | 190,058 |
| 199 – 219 | 268,004 | 207,300 | 139,302 |
| 220 – 240 | 199,404 | 150,569 | 101,632 |
| 241 – 261 | 141,008 | 105,232 | 70,639 |
| 262 – 282 | 100,747 | 73,121 | 48,550 |
| 283 – 303 | 74,033 | 51,781 | 34,375 |

Supplemental Table 3. List of SNPs above the suggestive threshold. The column calving number describes the analysis, in which the SNP was significantly associated. * marks SNPs which exceeded the Bonferroni threshold in the GWAS of the respective calving number.

| **Chromosome** | **Position (bp)** | **SNP** | **Effect** | **p-value** | **Calving number** |
| --- | --- | --- | --- | --- | --- |
| 4 | 74454166 | ARS-BFGL-NGS-110471 | -0.00566 | 1.20e-05 | 2 |
| 5 | 59922301 | Hapmap27767-BTA-154179 | -0.00488 | 1.00e-05 | 2 |
| 5 | 104993098 | ARS-BFGL-NGS-32818 | 0.00505 | 3.00e-06 | 2 |
| 5 | 106066646 | ARS-BFGL-NGS-111020 | 0.00737 | 1.20e-05 | 3 |
| 5 | 106320490 | ARS-BFGL-NGS-5240 | -0.00741 | 1.70e-05 | 3 |
| 5 | 107090339 | ARS-BFGL-NGS-16163 | -0.00494 | 3.00e-06 | 2 |
| 5 | 107090339 | ARS-BFGL-NGS-16163 | -0.00710 | 8.00e-06 | 3 |
| 6 | 95768489 | BTA-77418-no-rs | 0.00849 | 1.20e-05 | 3 |
| 8 | 72861312 | BTB-00357677 | 0.00489 | 1.30e-05 | 1 |
| 11 | 28191703 | ARS-BFGL-NGS-39693 | -0.00560 | 3.00e-06 | 2 |
| 11 | 29904900 | ARS-BFGL-NGS-118268 | -0.00510 | 2.00e-06 | 2, 1* |
| 11 | 29929724 | ARS-BFGL-NGS-68010 | 0.00604 | 4.00e-06 | 2 |
| 11 | 29977957 | ARS-BFGL-NGS-86581 | 0.00241 | 5.00e-06 | 1, 2* |
| 11 | 30804812 | BTB-00489432 | -0.00766 | 7.00e-06 | 3, 2* |
| 11 | 30916713 | ARS-BFGL-NGS-82216 | -0.00738 | 1.70e-05 | 3, 2* |
| 11 | 31004983 | ARS-BFGL-NGS-83866 | -0.00237 | 3.00e-06 | 1, 3* |
| 11 | 31222933 | ARS-BFGL-NGS-101601 | 0.00596 | 2.00e-06 | 2 |
| 11 | 31274292 | Hapmap45323-BTA-90907 | 0.00731 | 1.80e-05 | 3, 1* |
| 11 | 31477914 | ARS-BFGL-NGS-117672 | 0.00226 | 6.00e-06 | 1 |
| 11 | 31529943 | BTB-00468069 | -0.00644 | 2.00e-06 | 2 |
| 11 | 31609905 | BTB-00468020 | -0.00647 | 2.00e-06 | 2 |
| 11 | 32879031 | BTB-00471219 | -0.00562 | 4.00e-06 | 2 |
| 11 | 85937536 | Hapmap38876-BTA-109334 | -0.00482 | 1.80e-05 | 2 |
| 11 | 85972950 | BTA-120876-no-rs | -0.00495 | 2.00e-06 | 2 |
| 11 | 86020099 | ARS-BFGL-NGS-108297 | -0.00509 | 2.10e-05 | 2 |
| 11 | 86216179 | ARS-BFGL-NGS-115957 | 0.00464 | 1.00e-05 | 2 |
| 11 | 88765206 | ARS-BFGL-NGS-68659 | 0.00479 | 9.00e-06 | 2 |
| 16 | 25969288 | ARS-BFGL-NGS-102634 | -0.00541 | 6.00e-06 | 2 |
| 17 | 17642928 | ARS-BFGL-NGS-69762 | -0.00747 | 2.20e-05 | 3 |
| 23 | 29734356 | ARS-BFGL-NGS-109612 | 0.00396 | 1.10e-05 | 1 |
| 24 | 30461796 | Hapmap60714-rs29019480 | 0.00718 | 2.10e-05 | 3 |
| 24 | 30486009 | ARS-BFGL-NGS-115509 | 0.00502 | 8.00e-06 | 2 |
| 24 | 30532388 | ARS-BFGL-NGS-96695 | 0.00502 | 8.00e-06 | 2 |
| 24 | 30532388 | ARS-BFGL-NGS-96695 | 0.00721 | 1.80e-05 | 3 |
| 25 | 19902652 | ARS-BFGL-NGS-114006 | -0.00649 | 2.00e-05 | 3 |
| 25 | 20642417 | ARS-BFGL-NGS-111246 | 0.00734 | 2.00e-06 | 3 |
| 25 | 20683225 | ARS-BFGL-NGS-42285 | -0.00448 | 5.00e-06 | 2, 3* |
| 25 | 21117700 | BTB-00900391 | -0.00728 | 2.30e-05 | 3 |
| 25 | 21910606 | ARS-BFGL-NGS-60954 | 0.00572 | 3.00e-06 | 2 |
| 25 | 21910606 | ARS-BFGL-NGS-60954 | 0.00866 | 3.00e-06 | 3 |
| 25 | 25950148 | ARS-BFGL-NGS-115045 | 0.00737 | 4.00e-06 | 3 |
